# Determination of Mucoadhesion of Polyvinyl Alcohol Films to Human Intestinal Tissue

**DOI:** 10.3390/pharmaceutics15061740

**Published:** 2023-06-15

**Authors:** Laura Müller, Christoph Rosenbaum, Adrian Rump, Michael Grimm, Friederike Klammt, Annabel Kleinwort, Alexandra Busemann, Werner Weitschies

**Affiliations:** 1 Department of Biopharmaceutics and Pharmaceutical Technology, Institute of Pharmacy, University of Greifswald, Felix-Hausdorff-Str. 3, 17487 Greifswald, Germany; 2 Department of General, Visceral, Thoracic and Vascular Surgery, Greifswald University Medicine, Ferdinand-Sauerbruch-Str., 17457 Greifswald, Germany

**Keywords:** mucoadhesion, site-specific application, intestinal application, ex vivo measurements, human intestinal mucosa

## Abstract

The absorption of drugs with narrow absorption windows in the upper small intestine can be improved with a mucoadhesive drug delivery system such as enteric films. To predict the mucoadhesive behaviour in vivo, suitable in vitro or ex vivo methods can be performed. In this study, the influence of tissue storage and sampling site on the mucoadhesion of polyvinyl alcohol film to human small intestinal mucosa was investigated. Tissue from twelve human subjects was used to determine adhesion using a tensile strength method. Thawing of tissue frozen at −20 °C resulted in a significantly higher work of adhesion (*p* = 0.0005) when a low contact force was applied for one minute, whereas the maximum detachment force was not affected. When the contact force and time were increased, no differences were found for thawed tissue compared to fresh tissue. No change in adhesion was observed depending on the sampling location. Initial results from a comparison of adhesion to porcine and human mucosa suggest that the tissues are equivalent.

## 1. Introduction

An ideal drug substance should be absorbed uniformly throughout the small intestine. Some drugs are poorly absorbed due to narrow absorption areas, also known as absorption windows. These are usually located in the upper part of the small intestine. Poor absorption may be caused by specific transport mechanisms such as active transport or active excretion. Several drug delivery approaches have been developed to overcome this challenge, such as mucoadhesive films, which can be a highly beneficial drug delivery system (DDS) for site-specific applications, such as in the upper intestine. Examples of drugs that are only absorbed in the upper small intestine are furosemide [1], acyclovir [2,3] and gabapentin [4]. Other possible drugs that could benefit from prolonged residence time through mucoadhesion are therapeutic peptides and proteins [5,6]. These macromolecules mostly have very low oral bioavailability due to their high molecular weight and vulnerable structure. The specific amino acid sequence essential for drug activity can be destroyed by the chemical, physical and proteolytic nature of the gastrointestinal tract [7]. An ideal DDS for peptides and proteins should protect and preserve the drug structure and release it at the highly vasculated specific absorption site [8].

To predict the adhesion of the dosage form in vivo during formulation development, appropriate in vitro methods can be useful. In vitro methods have the advantage of good reproducibility and avoidance of biological tissues. Many biomimetic materials have been described in literature to mimic and replace tissue. They include, for example, simple hydrogels such as gelatin [9] or agar gels [10] and more complex hydrogels such as HEMA-AGA hydrogels [11] or mucin compacts [12]. The disadvantage of these biomimetic materials is that they may not adequately represent the inter-individual variability of ex vivo and in vivo studies. This can lead to biased prediction of in vivo behavior by in vitro methods. Therefore, ex vivo methods using tissue can be very helpful to get an idea of the variability in vivo. Ideally, the tissue used should represent as closely as possible the application site of the DDS under development.

Tissues from animal sources are mainly used as ex vivo substrates, such as chicken pouch [13], porcine tissue [14] or bovine tissue [15]. Although animal tissues are often used in ex vivo studies, there is the ethical drawback that animals have to be slaughtered to obtain the tissue. Along with these ethical concerns, the choice of suitable animal tissue is another issue. When it comes to mucoadhesion studies in the small intestine, rodents are known to be poor model animals. Not only the anatomy and physiology have been found to be different from humans [16,17] but also the pH and water content [18]. Therefore, large animal models such as pigs are often used to study the small intestine. However, although the pig anatomy is quite similar to that of humans, there are still some differences. Mucus thickness and composition are known to influence mucoadhesion [19]. In pigs, the average thickness of the small intestinal mucus is about 26 to 31 µm [19], whereas in humans the gastroduodenal mucus layer is of variable thickness [20]. These differences may affect mucoadhesion and the in vitro-in vivo correlation of mucoadhesion studies. Therefore, the ideal tissue for mucoadhesion studies is potentially human tissue. Patients taking medicines are usually elderly people suffering from more than one disease [21]. Their gastrointestinal tract may further differ from that of animals used in animal models. Theoretically, tissue from the target patient population should ideally be used to obtain the most predictive results.

Despite the choice of tissue, tissue preparation and storage may also affect the outcome of studies. In previous studies, mucoadhesion was found to be higher on thawed porcine small intestine tissue than on fresh tissue [22]. As these results may not be applicable to human tissue, further mucoadhesion studies on human small intestinal mucosa are needed. To the best of our knowledge, there is no ex vivo mucoadhesion study on human intestinal tissue. In this study, several questions will be addressed, the first of which is whether tissue storage has an effect on mucoadhesion in two different test setups. Secondly, the effect on the sampling site was investigated. Finally, a comparison was made with results on porcine tissue obtained in previous studies [22] using the same methodology. The results should indicate that the choice and storage of the tissue and the experimental design of each mucoadhesion study are very important variables that need to be investigated in order to understand the underlying mechanisms of mucoadhesion and to achieve predictive results for respective DDS.

## 2. Materials and Methods

### 2.1. Study Materials

The water-soluble polyvinyl alcohol quality EMPROVE^®^ ESSENTIAL PVA 18–88 (PVA 18–88, M_w_ ≈ 96,000 g/mol, Merck KGaA, Darmstadt, Germany) with a degree of hydrolysis of 88% was used as the mucoadhesive polymer for the preparation of the mucoadhesive films. Anhydrous glycerol (AppliChem GmbH, Darmstadt, Germany) was used as a plasticizer. The chemicals were dissolved in demineralized water.

### 2.2. Preparation of Mucoadhesive Films

Mucoadhesive films were prepared using the solvent casting technique on the day before the planned surgery. A total of 80.00 g demineralised water and 2.00 g anhydrous glycerol were mixed on a magnetic stirring plate (IKA^®^ RCT basic, IKA^®^-Werke GmbH & CO. KG, Staufen, Germany) at 250 rpm. Then, 18.00 g ground PVA 18–88 was added at 500 rpm. The dispersion was heated to 85 °C under continuous magnetic stirring at 150 rpm for 1 h until a clear solution was obtained. The solution was centrifuged at 4400 rpm for 15 min to remove air bubbles (Centrifuge 5702 R, Eppendorf SE, Hamburg, Germany). The solution was cast on a liner at 12.0 mm/s with a coating knife set to 1000 µm (mtv messtechnik oHG, Erftstadt, Germany) using an automatic coating bench (Automatic Precision Film Applicator CX4, mtv messtechnik oHG, Erftstadt, Germany). The cast film was dried at room temperature.

### 2.3. Study Participants

A positive ethical vote was obtained from the Ethics Committee of the University Medicine of Greifswald for the mucoadhesion study on human tissue (Ethical Protocol No. BB 027/21, date of approval: 2 March2021). A total of 13 patients (10 male, 3 females; BMI = 24.5 ± 5.5 kg/m^2^) aged 36 to 84 years (68 ± 13 years) was included. The patients suffered from various diseases of the gastrointestinal tract, such as cancer, sigmoid diverticulitis or Crohn’s disease. The operations during which the samples for the study were taken were directly related to these diseases. Written informed consent was obtained from all subjects and included information about the tissue sampling, the experimental plan, the handling of personal data and the data protection laws of Germany. During medically necessary surgery for Whipple procedure (n = 4), right hemicolectomy (n = 4), ileostomy (n = 4) or pancreatectomy (n = 1), a portion of healthy small bowel was also removed for technical reasons. This tissue was the proximal jejunum (n = 5) or the distal ileum (n = 8). In addition to demographic data, premedication data were also collected from the study participants.

### 2.4. Mucoadhesion Study

Mucoadhesion was determined using the same texture analysis method described in a previous study [22]. Briefly, a texture analyser (TA plus, AMETEK Lloyd Instruments Ltd., Hampshire, UK) equipped with a 10 N load cell was used to measure the maximum detachment force (F_max_) and the work of adhesion (WoA). Circular pieces (d = 14 mm, A ≈ 1.54 cm^2^) of the PVA films were punched out using a punching iron. The films were attached to the upper probe using double-sided adhesive tape (tesa^®^ Doppelseitiges Klebeband universal, tesa SE, Hamburg, Germany). The tissues were placed on the lower base of the apparatus. They were collected at the time of removal during surgery and transported to the laboratory in a polystyrene cooler filled with ice. To avoid direct contact, a bag filled with water was placed between the tissue placed in another bag and the ice. The time between the collection of the samples and the start of the experiments was a maximum of 30 min. The intestinal tissue was cut into four pieces, two of which were used immediately. The other two were placed in sealed PE bags and frozen at −20 °C in a freezer. After one week of storage, the tissues in the PE bags were thawed in a water bath at 37 °C.

F_max_ and WoA were measured in two settings based on a previous study [22]. In brief, a standard setting (setting A) was used as a starting point to investigate the influence of contact force, contact time and withdrawal speed on the mucoadhesion of PVA films to agar/mucin gels. Setting A was chosen on the basis of literature values. A low contact time and low contact force were used. In the following investigations, setting B was found to be the best compromise between the highest F_max_ and WoA and gel integrity. The experimental setups for both are described in Table 1. Each setting was performed on fresh and thawed tissue. The upper probe with the polymer film was lowered at a constant speed to the tissue from a distance of 5 cm until a specified contact force was detected. The probe remained in this position during the contact time and was then removed at a defined withdrawal speed. During removal from the tissue, load and machine extension were measured using NEXYGEN Plus software (AMETEK Lloyd Instruments Ltd., Hampshire, UK).

### 2.5. Statistical Analysis

F_max_ and WoA were calculated using Microsoft^®^ Excel^®^ 2019 (Microsoft Corporation, Redmond, WA, USA) and reported as individual data and medians. F_max_ was the maximum force measured during film detachment. WoA describes the area under the curve (AUC) and was calculated using the linear trapezoidal rule. Statistical analysis was performed using GraphPad Prism 5 (v. 5.01; GraphPad Software, Boston, MA, USA). F_max_ and WoA were tested for normal distribution using the D’Agostino and Pearson omnibus normality test. If the data were normally distributed and paired (e.g., derived from same subject), a paired t-test was used. Data that were not normally distributed were compared non-parametrically. A Wilcoxon signed rank test was used for paired data and a Mann-Whitney U test was used for unpaired data.

## 3. Results

A total of 13 subjects was initially part of the study. One subject (female, age = 84 years, BMI: 18 kg/m^2^) had to be excluded during the study because the amount of tissue removed for clinical reasons of the main indication was too small to perform mucoadhesion measurements with a sufficient number of samples. Data from this subject are excluded below. The demographics of the subjects are shown in Table 2.

In the remaining 12 subjects, both settings were to be performed on fresh and thawed tissue. In four subjects the tissue was too small to try both settings. Therefore, setting A with lower contact force, contact time and withdrawal speed was preferred on fresh and thawed tissue. The data have been checked for normal distribution. As not all data were normally distributed, a Gaussian distribution was not assumed for statistical comparison. The individual medians of the subjects can be found in Table S1.

### 3.1. Processing of the Tissue

The intestinal segments were divided into four parts, two of which were frozen and thawed for the experiments after a one-week storage period, and the other two were used fresh. As setting A was the preferred setting, a comparison of fresh and thawed tissue could be made for all 12 subjects.

**Figure 1:**
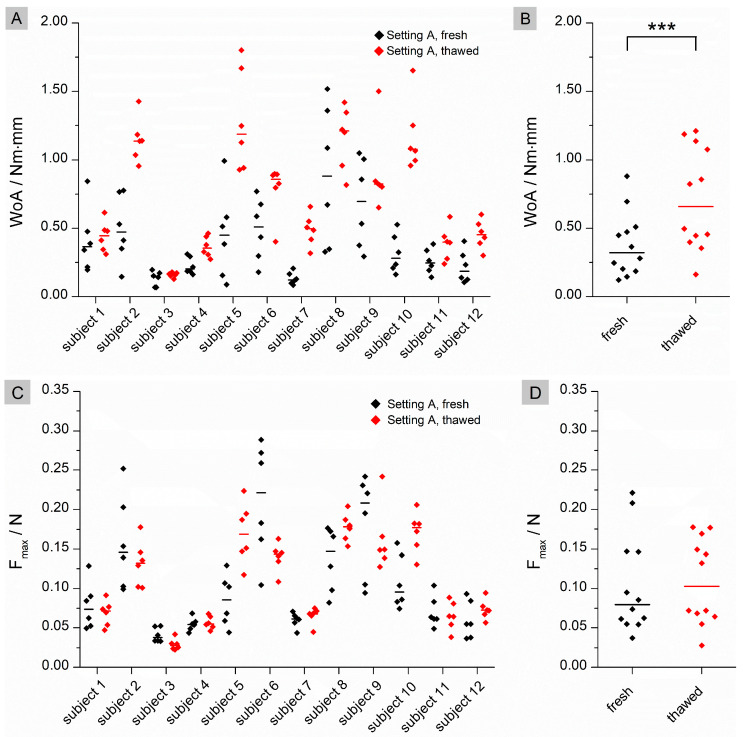
Results for Setting A. (**A**): Individual data of the calculated WoA (Nm × mm) with median (n = 12); black: fresh tissue; red: thawed tissue; (**B**): pooled medians of all subjects with median line; (**C**): Individual data of the calculated F_max_ (N) with median (n = 12); black: fresh tissue; red: thawed tissue; (**D**): pooled medians of all subjects with median line. Significant difference of WoA and F_max_ was checked by using a Wilcoxon signed rank test: *** (*p* < 0.001).

Significant differences were found for WoA (*p* = 0.0005) in setting A (Figure 1), whereas no significant difference was found for F_max_ (*p* = 0.6221). For individual data, WoA was higher on thawed tissue than on fresh tissue. No clear trend can be seen for F_max_.

**Figure 2:**
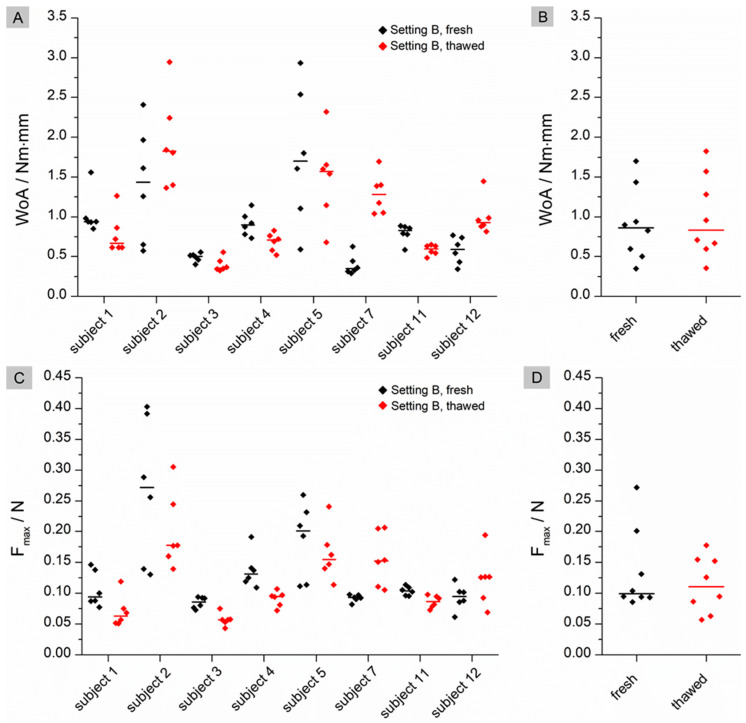
Results for Setting B. (**A**): Individual data of the calculated WoA (Nm × mm) with median (n = 8); black: fresh tissue; red: thawed tissue; (**B**): pooled medians of all subjects with median line; (**C**): Individual data of the calculated F_max_ (N) with median (n = 8); black: fresh tissue; red: thawed tissue; (**D**): pooled medians of all subjects with median line. Significant difference of WoA and F_max_ was checked by using a Wilcoxon signed rank test.

For setting B, where a higher contact time and force are applied, no significant differences can be found for either WoA (*p* = 0.7422) or F_max_ (*p* = 0.3125) (Figure 2). In contrast to setting A, no trend can be seen in the individual data for either of the calculated mucoadhesion values. Overall, the measured and calculated results were higher in setting B than in setting A.

### 3.2. Comparison of Different Test Settings

Fresh and thawed tissues were also compared for both settings to investigate the influence of the test parameters. Setting A used a lower contact time, lower contact force and lower withdrawal speed. Only data from participants who were able to use both settings were included in the comparison, resulting in eight measurements.

**Figure 3:**
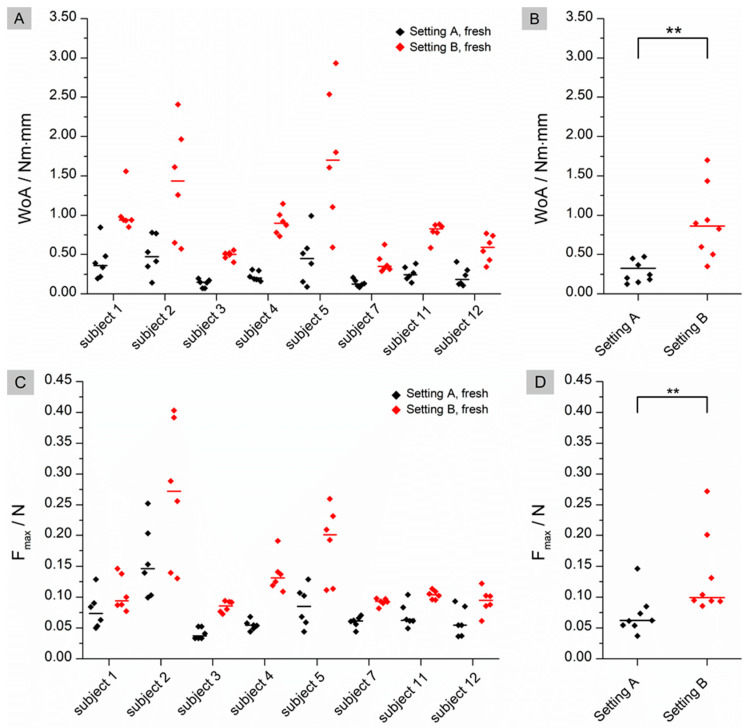
Comparison of the results for Setting A and B on fresh tissue. (**A**): Individual data of the calculated WoA (Nm × mm) with median (n = 8); black: Setting A; red: Setting B; (**B**): pooled medians of all subjects with median line; (**C**): Individual data of the calculated F_max_ (N) with median (n = 8); black: Setting A; red: Setting B; (**D**): pooled medians of all subjects with median line. Significant difference of WoA and F_max_ was checked by using a Wilcoxon signed rank test: ** (*p* < 0.01).

As shown in Figure 3, the fresh tissue showed significant differences between setting A and setting B for WoA (*p* = 0.0078) and F_max_ (*p* = 0.0078). For both WoA and F_max_, the median of each individual data set was significantly higher in setting B than in setting A.

**Figure 4:**
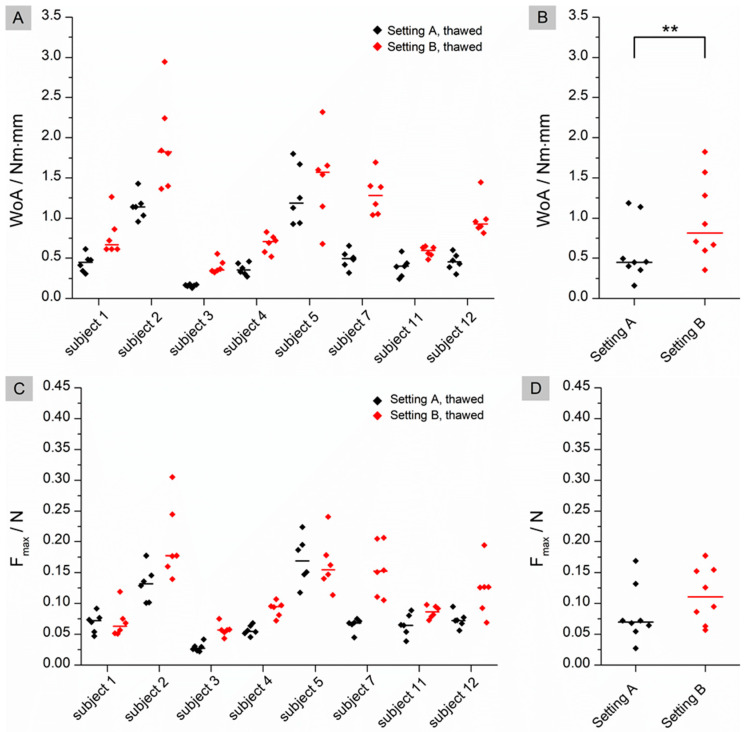
Comparison of the results for Setting A and B on thawed tissue. (**A**): Individual data of the calculated WoA (Nm × mm) with median (n = 8); black: Setting A; red: Setting B; (**B**): pooled medians of all subjects with median line; (**C**): Individual data of the calculated F_max_ (N) with median (n = 8); black: Setting A; red: Setting B; (**D**): pooled medians of all subjects with median line. Significant difference of WoA and F_max_ was checked by using a Wilcoxon signed rank test: ** (*p* < 0.01).

The same comparison was made for thawed tissue. As shown in Figure 4, statistically significant differences were also found for WoA (*p* = 0.0078), but not for F_max_ (*p* = 0.3828).

### 3.3. Sampling Location

Another issue was the importance of the sampling site, as there may be differences in adhesion in the proximal jejunum compared with the distal ileum. A Mann-Whitney U test was performed as the data were not paired and the number of samples was too small to assume a normal distribution. The test was performed on fresh and thawed tissue for setting A only, as the number of samples in this case was 12. No statistical differences were found for either WoA (*p*_fresh_ = 0.5303; *p*_thawed_ = 0.2020) or F_max_ (*p*_fresh_ = 0.2677; *p*_thawed_ = 0.1490).

### 3.4. Comparison of Mucoadhesion on Porcine Versus Human Intestinal Tissue

**Figure 5:**
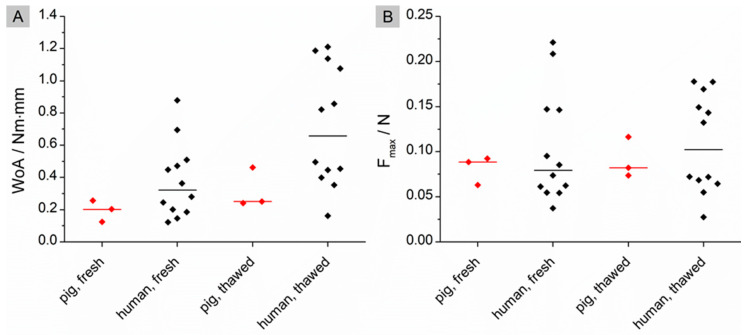
Comparison of the results for Setting A on fresh tissue of pigs (n = 3) and humans (n = 12). (**A**): Pooled medians of the calculated WoA (Nm × mm) with median line; red: pig; black: human; (**B**): Pooled medians of the calculated F_max_ (N) with median line; red: pig; black: human. Significant difference of WoA and F_max_ was checked by using a Mann-Whitney U test.

The data obtained in this study were compared with those of a previous study carried out on porcine small intestine tissue [22]. In the previous study, the identical test setup A was used to measure mucoadhesion. The only difference, apart from the origin of the tissue, was that the number of samples was much smaller (n = 3) compared to the new study (n = 12). Cleaned porcine tissue was used as a reference because, unlike the participants’ tissue, it was not free of food residues due to the surgical specifications. As a result, the statistical analysis presented below may only give an indication of the difference. A Mann-Whitney U test was performed to evaluate possible differences in WoA and F_max_ between fresh and thawed tissue. The results are shown in Figure 5.

No significant differences could be found for the WoA (*p*_fresh_ = 0.2790; *p*_thawed_ = 0.1296) nor the F_max_ (*p*_fresh_ = 0.9425; *p*_thawed_ = 0.9425). The data presented in Figure 5A indicate a trend towards a slightly higher WoA on fasted human tissue compared to washed porcine tissue. However, the sample numbers of porcine tissues are too small to state this with certainty.

## 4. Discussion

**Figure 6:**
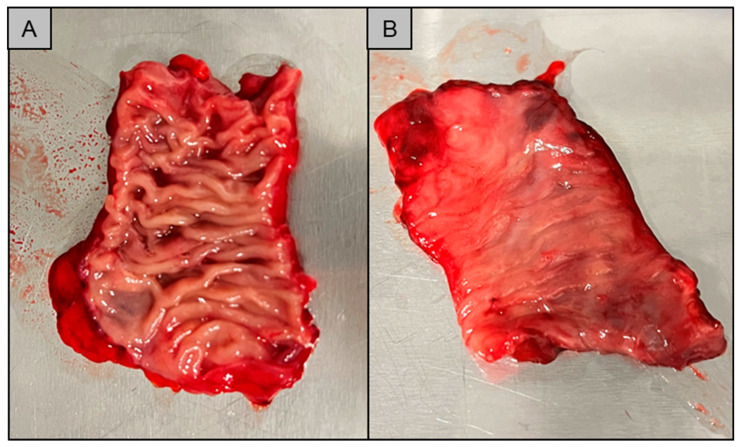
(**A**): Fresh tissue; (**B**): thawed tissue after a storage time of 7 days at T = −20 °C in a freezer.

Ex vivo mucoadhesion measurements of PVA-films on human small intestine tissue show that F_max_ and WoA are highly variable inter-individually and intra-individually. Possible influences on the measurement results were investigated. Statistical analysis of the mucoadhesion values in two settings and on tissues prepared in different ways showed that the WoA appears to be sensitive to storage and test parameters. WoA was significantly higher on thawed tissue in setting A, where a lower contact force is applied for a shorter time, but surprisingly not when a higher force is applied for a longer contact time, as in setting B. If tissue is frozen without a cryoprotectant, ice crystals may form. This depends on the rate at which the tissue is frozen. A slow freezing rate often results in the formation of sharp crystals that can damage tissue cells by perforating them [23]. In addition, cells can be further damaged by the osmotic pressure that can result from ice formation [23]. Signs of possible tissue damage were observed after storage of the respective tissue samples. The appearance of the tissue changed during storage. The macrostructure of the tissue appeared flatter than in the fresh condition. There was also some leakage of fluid from the tissue as can be seen in Figure 6.

The flattened structure of thawed tissue may explain the higher observed WoA. When a low force is applied in the fresh state, the mucoadhesive film may not be in contact with the entire tissue due to the macroscopically visible folded structure. The contact forces of 0.1 N and 0.35 N correspond to biorelevant pressures of 6.5 mbar and 22.7 mbar, respectively. These are within the physiological range of the small intestine as determined in telemetric studies with the SmartPill [24]. Higher contact forces may result in a flatter structure due to tissue compression and therefore more even contact between the film and the mucosa. As a result, WoA may increase when higher forces are applied (setting A versus setting B) or when the tissue loses structure due to thawing. No statistical differences can be found for WoA on fresh versus thawed tissue in setting B. A possible reason for this finding could be that not only the macrostructure but also the microstructure of the mucus changes during storage, as observed by Hägerström et al. [25]. Negatively charged glycoproteins called mucins, which make up approximately 0.5–5% of mucus [26], play an essential role in mucoadhesion. Mucoadhesive polymers can bind to mucins either through chemical bonds, such as ionic, covalent or secondary bonds, or through physical bonds. These include interpenetration and entanglement of polymer structures and mucin chains [27]. Polyvinyl alcohol, which was used in our study, is a non-ionic polymer. This group of polymers is known to bind to mucus through secondary chemical bonds such as hydrogen bonds and chain entanglements. Typically, the mucoadhesion of non-ionic polymers is lower than that of cationic polymers such as chitosan, which bind by electrostatic attraction to negatively charged mucins [28]. If the structure of the mucus changes during freezing and thawing [29], it may loosen, resulting in a looser structure that potentially facilitates interpenetration and chain entanglement. This may have a positive effect on the mucoadhesion of non-ionic polymers, as observed for thawed tissue in setting A (Figure 1B). The influence of a higher contact force (setting B) appears to have a greater effect on mucoadhesion than the thawing process, as there are no statistical differences between fresh and thawed tissue in setting B. However, the loss of the microstructure of mucus may influence the adhesion of charged polymers. When mucus hydrogels are frozen and thawed, there is a phase separation between the aqueous phase and the hydrogel former, resulting in a concentration of mucins. This in turn can lead to an increased number of possible electrostatic interactions, resulting in a higher mucoadhesive work. In contrast to WoA, F_max_ is not influenced by storage (Figure 1D and Figure 2D). The question therefore arises as to whether WoA or F_max_ is the better surrogate for the measurement of mucoadhesion. In their paper, das Neves et al. [30] discussed whether WoA or F_max_ is more suitable for evaluating the mucoadhesion of semi-solids to bovine vaginal mucosa. WoA represents the sum of all adhesive forces, whereas F_max_ represents only the peak force during detachment. Therefore, the authors consider WoA to be the more accurate parameter for mucoadhesion. Da Silva et al. [12] also confirmed in their work that the WoA is more sensitive to changes in the test parameters and therefore the better surrogate for mucoadhesion studies in texture analyser studies. These results are confirmed by our study. For future mucoadhesion measurements with the texture analyser, it should be noted that both the test parameters and, in particular, the storage of the tissues have an influence on the measurement results, making it difficult to compare different studies.

In addition to the storage and test setup, the influence of the sampling site was investigated. No statistical differences were found between the results obtained from the proximal jejunum and the distal ileum. Another patient-specific parameter that may influence the results of the study is the amount of aqueous medium (e.g., mucus and/or bile acid) present on the tissue. A higher amount of water can cause faster hydration of the solid polymer in the mucoadhesive film. This effect is advantageous in the contact stage, as chain disentanglement of the former solid polymer occurs upon hydration [31]. The detrimental effect begins as soon as the polymer hydrogel is diluted. If the amount of water in the polymeric gel becomes too high, the cohesiveness of the gel will decrease. This results in a failure of adhesion within the gel as the test preparation is detached from the mucosa which is represented by lower values for F_max_ and WoA. To minimise the influence of intestinal fluids, the tissues can be washed [32] or wettened with a specified amount of liquid [33] prior to the experiment. The disadvantage of these methods is that the tissues may no longer represent the actual in vivo state.

Another patient-related factor to be considered is age. Intestinal morphology does not appear to change in older people [34,35]. There is some evidence that there may be changes in the structure of mucus with age. Elderman et al. [36] reported that the age of mice can influence the thickness of their colonic mucus, with older mice having a thinner layer of mucus compared to young mice. As mentioned above, mucus thickness may influence mucoadhesion, so it would be interesting to investigate age-related changes in mucus in humans. Other important changes that occur with ageing are increased illness and polymedication [21]. Drugs and inflammatory bowel diseases could also affect mucoadhesion, as they can affect pH and mucus [37]. It is important to note that there are many factors that can influence mucoadhesion in vivo, especially in the elderly. These factors are less likely to have a visible effect on ex vivo mucoadhesion studies as their influence may be small. However, when it comes to in vivo performance, they should be considered.

The final point evaluated in our study was the comparison of mucoadhesion to porcine versus human small intestinal tissue. The data for porcine tissue were taken from a previous study carried out in our laboratory [22] under the same conditions. It should be noted that the results of this comparison can only indicate a possible trend, as the number of samples for the porcine tissue was too small. This is related to the fact that the porcine experiments were aimed at a broader screening with more different setups and variables, thus limiting the sample size of measurements comparable to human ex vivo measurements from this study. No significant differences were found for either WoA or F_max_ on fresh and thawed porcine or human tissue. The individual data may suggest that the WoA is slightly higher on human mucosa compared to porcine mucosa, especially when thawed tissue is used. This again might lead to the conclusion that the WoA is the better surrogate for mucoadhesion as it seems to be more sensitive to changes in the tested mucosal sample.

Pigs are often used as model animals for studies involving the gastrointestinal tract because their gastrointestinal physiology is very similar to that of humans [16]. In addition, the availability of tissues is usually good, as pigs are common farm animals, and intestinal tissues are most often slaughterhouse waste. Jackson and Perkins reported that the mucoadhesion of cholestyramine on porcine gastric mucosa was found to be higher than with human mucosa [38]. They explained this result with a possibly thicker mucus layer in pigs compared to humans. This is contrary to the results obtained in our study, but the limited number of porcine tissue samples and a different mucosa may influence the outcome of these studies.

## 5. Conclusions

The purpose of this ex vivo study was to highlight the inter-individual variability of mucoadhesion to human small intestine tissue. The study data showed the range of individual results, highlighting the high variability of biological materials. The results show that an ideal mucoadhesive DDS should be able to demonstrate good adhesion despite the high inter-individual variability. PVA mucoadhesive films were used to investigate the test-related factors influencing this variability.

Storage is an important factor influencing the study results and should be considered when performing a mucoadhesion test. The WoA seems to be more sensitive to the storage of tissue when a force of 0.1 N is applied for 60 s. The effect on F_max_ is less pronounced. No statistical differences for both can be found if a higher force of 0.35 N is applied for 180 s. Comparing setting A (lower force and contact time) to setting B (higher force and contact time) shows that there is a significant difference in the measurement results of WoA and F_max_ on fresh tissue. Again, no difference was found on thawed tissue for the F_max_. Therefore, WoA is assumed to be the better surrogate for mucoadhesion. The results show that the adhesion is dependent on both the test setup and the sample preparation. An ideal test setup and storage of the sample to which the dosage form is to adhere must be individually tested prior to each test.

Although the data available were limited, a comparison of mucoadhesion on porcine and human mucosa was made. The initial impression is that the two tissues are comparable. If further studies confirm the hypothesis that porcine intestinal tissue could replace human intestinal tissue, this would facilitate ex vivo mucoadhesion studies. Tissue of animal origin can be obtained in larger quantities and without the regulatory requirements of ex vivo human tissue studies.

Despite the test-related factors, the sampling site was examined as a patient-related factor. No difference was found between proximal jejunum and distal ileum. Other patient-related factors need to be investigated in the future, as mucoadhesion is a complex phenomenon and the understanding of all factors affecting mucoadhesion in vivo is still limited. Gender, mucus thickness, gastrointestinal fluids, diseases and medications may be other parameters to consider. A larger number of subjects would be needed to gain a deeper understanding of the physiological effects on mucoadhesion and to design an ideal DDS that is minimally affected by these variables. A simple way to address the variability and allow comparability between different test devices, tissue preparations and possible new innovative delivery forms could be to measure adhesion against a simple and reproducible manufacturable standard, such as a polyvinyl alcohol film.

## Figures and Tables

**Table 1:** Instrument settings for Setting A and B.

Variable	Setting A	Setting B
contact force [N]	0.1	0.35
contact time [s]	60	180
withdrawal speed [mm/s]	0.5	1.0

**Table 2:** Demographic data of the remaining 12 study participants.

Parameter	Median (Range)	Mean ± SD
sex	m = 10; f = 2	m = 83%; f = 17%
age/y	70 (36–80)	67 ± 12
height/m	1.76 (1.59–1.87)	1.74 ± 0.09
weight/kg	75.6 (43.0–105.0)	75.6 ± 16.8
BMI/kg/m^2^	23.9 (16.4–37.6)	25.0 ± 5.4

## Data Availability

Data are available within the paper and its supplementary material.

## Supplementary Materials

The following supporting information can be downloaded at: https://www.mdpi.com/article/10.3390/pharmaceutics15061740/s1, Table S1. Individual medians of F_max_ (N) and WoA (Nm × mm) of all subjects in both test setups (setting A and B) and different tissue preparation. All tests were performed in six replicates.
