# Supplementary material for: Determination of Mucoadhesion of Polyvinyl Alcohol Films to Human Intestinal Tissue
Source: Pharmaceutics. 2023 Jun 15;15(6):1740. doi: 10.3390/pharmaceutics15061740 (PMC10301857; doi:10.3390/pharmaceutics15061740)
Supplement: Supplementary file 1 [file pharmaceutics-15-01740-s001.zip › pharmaceutics-2441679-supplementary.pdf]

Table S1. Individual medians of F_{max} (N) and WoA (Nm·mm) of all subjects in both test setups (setting A and B) and different tissue preparation. All tests were performed in six replicates.

	**Setting A**				**Setting B**			
	fresh		thawed		fresh		thawed	
	**WoA**	**Fmax**	**WoA**	**Fmax**	**WoA**	**Fmax**	**WoA**	**Fmax**
	Nm·mm	N	Nm·mm	N	Nm·mm	N	Nm·mm	N
subject 1	0.3632	0.0737	0.4463	0.0718	0.9385	0.0942	0.6687	0.0627
subject 2	0.4713	0.1463	1.1374	0.1323	1.4356	0.2719	1.8220	0.1773
subject 3	0.1463	0.0374	0.1617	0.0275	0.5028	0.0860	0.3559	0.0570
subject 4	0.2012	0.0542	0.3529	0.0550	0.8966	0.1312	0.7055	0.0948
subject 5	0.4476	0.0853	1.1868	0.1691	1.7021	0.2009	1.5686	0.1546
subject 6	0.5098	0.2212	0.8566	0.1433	*	*	*	*
subject 7	0.1227	0.0613	0.4952	0.0684	0.3460	0.0930	1.2825	0.1527
subject 8	0.8794	0.1469	1.2107	0.1779	*	*	*	*
subject 9	0.6946	0.2082	0.8215	0.1491	*	*	*	*
subject 10	0.2801	0.0951	1.0752	0.1772	*	*	*	*
subject 11	0.2445	0.0624	0.3990	0.0646	0.8236	0.1039	0.5950	0.0867
subject 12	0.1859	0.0548	0.4539	0.0722	0.5933	0.0951	0.9258	0.1263

* For these subjects the tissue was too small to try both settings. Therefore. setting A with lower contact force. contact time and withdrawal speed was preferred on fresh and thawed tissue.
